# Vascular mimicry in cultured head and neck tumour cell lines

**DOI:** 10.1186/1758-3284-3-55

**Published:** 2011-12-23

**Authors:** Tahwinder Upile, Waseem Jerjes, Hani Radhi, Mohammed Al-Khawalde, Panagiotis Kafas, Seyed Nouraei, Holger Sudhoff

**Affiliations:** 1 The Ear Institute, University College London, London, UK; 2 Department of Head & Neck Surgery, Bielefeld Hospital, Germany; 3 Department of Head & Neck Surgery, The Professorial Unit, The Royal National Throat, Nose and Ear Hospital, London, UK; 4 UCLH Head and Neck Centre, London, UK; 5 Department of Surgery, University College London Medical School, London, UK; 6 Leeds Institute of Molecular Medicine, School of Medicine, University of Leeds, Leeds, UK; 7 Oral and Maxillofacial Surgery Unit, AL-Mustansirya University, Baghdad, Iraq; 8 Oral and Maxillofacial Surgery Unit, Royal Medical Services, Amman, Jordan; 9 Department of Oral Surgery and Radiology, School of Dentistry, Aristotle University, Thessalonica, Greece

## Abstract

**Introduction:**

Vascuologenesis is the de novo establishment of blood vessels and vascular networks from mesoderm-derived endothelial cell precursors (angioblasts). Recently a novel mechanism, by which some genetically deregulated and aggressive tumour cells generate "micro-vascular" channels without the participation of endothelial cells and independent of angiogenesis, has been proposed. This has been termed "vasculogenic mimicry" and has implications beyond angiogenesis and adds another layer of complexity to the current concept for the generation of tumour micro-circulation. We suggest this is common phenomenon in head and neck squamous cell carcinoma (HNSCC) cell lines and other aggressive tumour cell lines. We present experimental evidence of vasculogenic mimicry in HNSCC cell lines and compare them with other tumours and a positive control vascular cell line.

**Materials and methods:**

The cell lines used were HUVEC, HN 2a, 2b (primary and metastatic tongue base squamous carcinoma cell line), HCT116 (colonic carcinoma cell line) and DU145 (prostate carcinoma cell line).

Pilot experiments were undertaken to assess growth of a bank of tumour cell lines on (growth factor reduced) matrigel (Sigma) with standard media (DMEM with 10% Fetal Calf Serum).

A functional growth assay was performed by preparing the appropriate cell suspension in serum free medium plated onto either bare plastic or a well pre-coated with growth factor reduced type 4 collagen analogues.

Phase contrast photomicrographs were taken at 4 hours and 24 hours. Image analysis was performed; particular features of interest were two dimensional area (surrogate of growth and migration), branch points and end point measurements (surrogate of intercellular complexity).

**Results:**

There were observable differences in growth of the cells on laboratory plastic and collagen matrix. Tumour cells formed capillary like networks similar to HUVEC cells. Metastatic HNSCC cells lines were found to have vasculogenic properties similar to HUVEC cell lines when compared to cell lines from their corresponding primary tumour. The endothelial growth factor antibodies used did not inhibit or stimulate cell growth when compared to control but did discourage vascular mimicry. Other tumour cell lines also displayed this property.

**Discussion:**

Tumour "vasculogenic mimicry" must still be regarded as a controversial issue whose existence is not proven. The clinical importance of this phenomenon however, is that it does explain the lack of complete efficacy of current anti-angiogenic treatments due to the added layer of complexity. It provides a feasible mechanism of early tumour vascular supply which can co-exist and incorporate with later angiogenic mechanisms. We suggest that "vasculogenic mimicry" maybe a common neoplastic phenomena which appears to also be dictated by the cells micro-environment. Its existence also suggests a further process that of the development of tumour mosaic vessels as the neo-vasculature integrates with the existing endothelial lined systems.

## Introduction

The growth of solid tumours is limited to the distance that oxygen, nutrients and waste products can diffuse (1-2 mm), thus malignancy tends to remain dormant at the size of 2-3 mm^3 ^in the absence of neo-vascularisation. Much attention has been focussed on the role of angiogenesis, i.e. the recruitment or co-option of new vessels into a tumour from pre-existing vessels such as capillaries and post-capillary venules. Currently, angiogensis is widely accepted as the mechanism by which tumours metastasize, however angiogenesis may not be the only mechanism by which tumours acquire a microcirculation. Maniotis et al. reported a novel mechanism by which some aggressive tumours acquire a blood supply and demonstrated the generation of micro-vascular channels by genetically deregulated and aggressive tumour cells without the participation of endothelial cells and independent of angiogenesis. This has been termed "vasculogenic mimicry" and has implications beyond angiogenesis and adds another layer of complexity to the current theoretical framework for the generation of tumour micro-circulation [1].

Vascuologenesis hence is the de novo establishment of blood vessels and vascular networks from mesoderm-derived endothelial cell precursors (angioblasts). In contrast, the expansion of the vasculature by angiogenesis is dependent on the generation of additional endothelial cells from pre-existing vascular beds, i.e. it is the source of the newly generated vascular lining or "endothelial cells" that distinguishes vasculogenesis from angiogenesis. There is no doubt that there is an overlap of mediators and signalling systems in these two systems but their roles may differ. Tumour cell plasticity is demonstrated by the ability of tumour cells to adopt a variety of phenotypes, including an endothelial phenotype [2,3] to allow survival. These findings emphasize the plasticity of malignant cells from advanced tumour progression stages, and they require more dynamic view of the metastatic cascade.

We need to understand how the malignant cells exert cooperation from the normal cells. It is hypothesized that both normal and malignant cells utilize the same molecules for invasion, but that differences in downstream signalling events allow the tumour cells to dominate over normal cells in the microenvironment. This "dominant plasticity" model of cancer metastasis takes into account the flexible response of malignant cells to micro-environmental pressures while maintaining dominance over the normal parenchymal and stromal cells.

Vasculogenic mimicry has now been described in a variety of tumours, i.e. ovarian and breast cancers, and choroidal Melanomas [2-4]. We hypothesize that vasculogenic mimicry is a common neoplastic phenomenon which appears to also be dictated by the cell's micro-environment (cell cytoskeletal interactions). Its existence also suggests a further process that of the development of tumour mosaic vessels as the neo-vasculature integrates with the existing endothelial lined systems. We present evidence of vasculogenic mimicry in head and neck cancer cell lines and compare them with other tumours and a positive control vascular cell line.

## Materials and methods

The cell lines used were HUVEC, HN 2a, 2b (primary and metastatic tongue base squamous carcinoma cell line), HCT116 (colonic carcinoma cell line) and DU145 (prostate carcinoma cell line). Cells were grown in culture flasks (Costar 75 cm^2 ^Vent Cap) in their appropriate growth media (DMEM with 10% Fetal Calf Serum, 5% CO^2^). Cells were grown to 75% confluence and serum starved for 24 to 48 hours before use. Experiments were carried out using the appropriate serum free medium for each cell line. Mycoplasma PCR assays were carried out to exclude infection.

**Figure 1:**
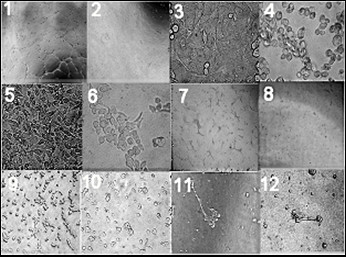
**Morphological examination of cell lines by phase contrast micrographs at 24 hours**. There appears an effect of substrate and antibody on cell line growth patterns. The cell lines used were (1) Huvec cells on matrigel (X200); (2) Huvec cells on matrigel with antibody (X200); (3) HN2a cells on plastic (X200); (4) HN2a cells on matrigel (X200); (5) HN2b cells on plastic (X200); (6) HN2b cells on matrigel (X200); (7) HN2b cells on matrigel (X100); (8) HN2b cells on matrigel with antibody (X100); (9) HCT116 cells on matrigel (X200); (10) HCT116 cells on matrigel with antibody (X200); (11) DU 145 cells on matrigel (X200); (12) DU145 cells on matrigel with antibody (X200).

Pilot experiments were undertaken to assess growth of a bank of tumour cell lines on (growth factor reduced) matrigel (Sigma) with standard media (DMEM with 10% Fetal Calf Serum). The cells were incubated at 37°C with 5% CO_2_. Phase contrast photomicrographs (Olympus systems) were taken at 4 hours and 24 hours. Image analysis was performed by using Image Pro Plus 4.0 computer image analysis software, particular features of interest were two dimensional area (surrogate of growth and migration), branch points and end point measurements (surrogate of intercellular complexity), (Figure 1).

A modified growth assay was undertaken whereby cells were grown to 75% confluence and plated directly upon a 96 well plate at a seeding density of 2*10^3 ^cells per well, and serum starved for 24 hours. The cells were then grown in serial dilutions of either pure antibody mixture or blood product or a combination for a further 48 hours. 100 μl of alkaline phosphates substrate (3 mg/ml) was then added to each well, incubated for 2 hours after which 50 μl of 1 M NaOH was added and the plate placed in a micro-plate reader and read at 405 nm wavelength.

A functional growth assay was performed by preparing the appropriate cell suspension in serum free medium plated onto either bare plastic or a well pre-coated with growth factor reduced matrigel [200 ul of matrigel per well (incubated for 30 minutes)] at a seeding density of 2*10^5 ^cells per well. Where appropriate, cells were pre-incubated for 1 hour with 10 ul of a mixture of 100 ul of each anti-receptor (anti-FLK1, anti-FLT1, anti-FLT4) and anti-ligand antibodies (anti-VEGFA, anti-VEGFC, anti-VEGFD). The fresh media or antibody in fresh media was added to make a final volume of 900 ul and the cells incubated for a further hour, after which a 100 ul of appropriate blood product (with or without antibody) was added. The cells were incubated at 37°C with 5% CO_2_. Phase contrast photomicrographs (Olympus systems) were taken at 4 and 24 hours. Image analysis was performed by using Image Pro Plus 4.0 computer image analysis software, particular features of interest were two dimensional area (which can be used as a surrogate of growth and migration), branch points and end point measurements (surrogate of intercellular complexity, i.e. number of capillaries), (Figure 1). All results are representative of at least two independent experiments carried out in triplicate. Statistical analysis was performed using Graph Pad Prism 4.0 software.

## Results

**Figure 2:**
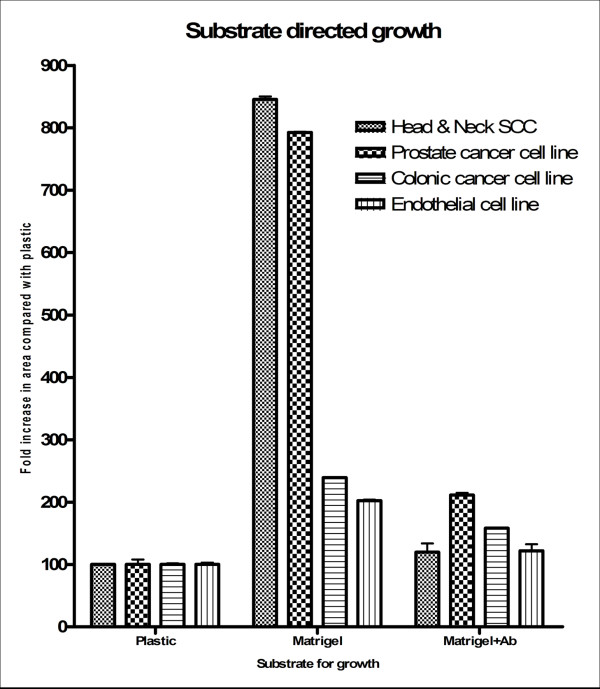
**Substrate directed growth of common tumour cell lines on matrigel, a collagen basement membrane analogue, resulted in significant growth and vascular channel formation when compared to growth on standard laboratory culture plastic, (P < 0.001)**. Furthermore the magnitude of this effect was reduced by the use of specific anti-endothelial antibodies, (P < 0.001). Graph showing fold increase in growth area compared to "standard" culture on laboratory Petri dishes. The Cell lines used were HN2b (head and neck SCC), DU145 (Prostate cancer), HCT116 (Colonic cancer) and HUVEC (Human Endothelial cells).

There were observable differences in growth of the cells on laboratory plastic and collagen matrix (P < 0.001), (Figure 2). Tumour cells formed the appearance of capillary-like networks similar to HUVEC cells (an endothelial cell line).

Metastatic cell lines (HN2B) were found to have vasculogenic properties similar to HUVEC cell lines when compared to cell lines from their corresponding primary tumour (HN2A). The clinical impact of this phenomenon will also be a function of tumour cell size enabling optimal stromal interaction as evinced by the growth of other tumour cell lines.

The anti-endothelial antibody was not cytotoxic to any of the cell lines investigated. The antibody, however, did discourage vascular mimicry significantly (P < 0.001). It may be hypothesized that the visible networks may not be tubes and not represent vascular channels but electron microscopy work suggests that they do [1].

## Discussion

**Figure 3:**
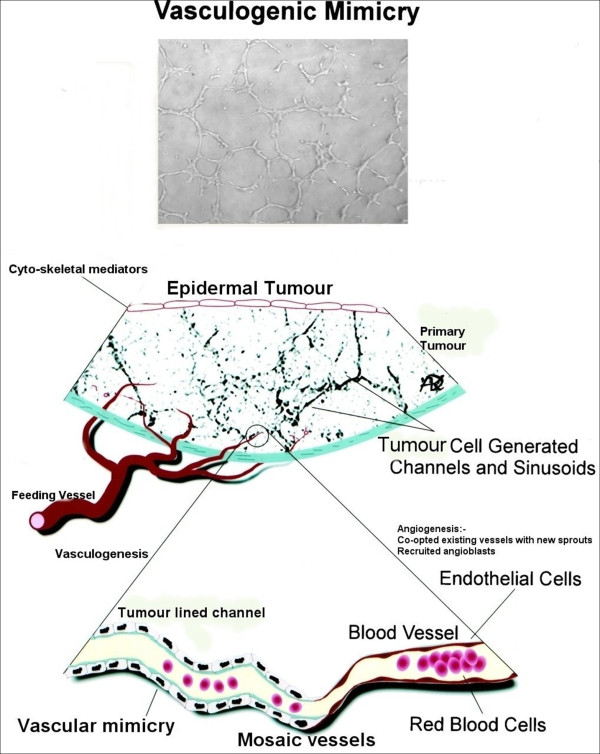
**Showing the hypothesis of "vasculogenic mimicry" in head and neck cell lines**. The upper part of the image shows vascular channel formation in a head and neck cell line. The lower part is a diagrammatic representation of a tumour lines blood vessel and its transition to a mosaic lined vessel (containing tumour and co-opted "angiogenic sprouts" from host endothelial cells) joining a conventional endothelial lined capillary of the systemic vasculature. This figure was adapted with permission and modified from: Folberg R, Hendrix MJ, Maniotis AJ. Vasculogenic mimicry and tumor angiogenesis. Am J Pathol. 2000 Feb;156(2):361-81.

Tumour "vasculogenic mimicry" (Figure 3) must still be regarded as a controversial issue whose existence is not proven. The clinical importance of this phenomenon however, is that it does explain the lack of complete efficacy of current anti-angiogenic treatments due to the added layer of complexity. It provides a feasible mechanism of early tumour vascular supply which can co-exist and incorporate with later angiogenic mechanisms. We suggest that "vasculogenic mimicry" maybe a common neoplastic phenomena (as seen in the tumour cells lines investigated) which appears to also be dictated by the cells micro-environment (cell cytoskeletal interactions). Its existence also suggests a further process that of the development of tumour mosaic vessels as the neo-vasculature integrates with the existing endothelial lined systems.

The process of angiogenesis consists of multiple, sequential and independent steps with a vista of positive and negative regulators of angiogenesis being involved [5]. However, the mechanism by which an aggressive tumour initially generates its own network of pseudo-vascular channels challenges the assumption that angiogenesis and related mechanisms are the only means by which a tumour acquires a blood supply [6]. Cell-cell interactions and adhesion molecules: linking proliferation, survival, and motility should not be overlooked in this cascade. The stromal microenvironment, in which neoplastic cells develop, profoundly influences many steps of cancer progression, including the ability of tumor cells to metastasize. These influences of the microenvironment are mediated, in large part, by bidirectional interactions (adhesion, survival, proteolysis, migration, immune escape mechanisms lymph-/angiogenesis, and homing on target organs) between epithelial tumour cells and neighbouring stromal cells, i.e. fibroblasts as well as endothelial and immune cells. Central to the metastatic process, i.e. the changing adhesive preferences of the cancer cells, from epithelial cells to fibroblasts and endothelial cells, that dictate their reciprocal interactions with the extracellular matrix (ECM) and neighbouring stromal cells [7].

There is a consensus that the microcirculation of aggressive tumours is complex, and depending on the time of observation, could consist of mosaic vessels (both tumour cells and endothelial cells), co-opted vessels and/or angiogenic vessels. There is also strong evidence for the existence of an intratumoral, tumour-cell-lined, ECM-rich, patterned network that can provide an extravascular fluid pathway, now known as the fluid-conducting meshwork. The entire microcirculation in aggressive tumours is suggested to be made up of a combination of these elements, and it is the result of destructive tumour growth and remodelling. Alternatively, the PAS- and laminin-rich, fluid-conducting meshwork could be an early survival mechanism for nutrient exchange and the release of fluid pressure. This meshwork could eventually be replaced by endothelial cells from nearby angiogenic vessels or from the bone marrow. The fluid-conducting meshwork might provide a site for nutritional exchange for aggressive tumours, and might therefore prevent necrosis of the tumour. Alternatively, it might be analogous to an oedematous inflammatory response, in which increased blood pressure leads to the escape of fluid along connective tissue pathways in intra-tissue spaces. The complex geometry of the laminin-containing ECM covering that encases the spheroidal clusters of tumour cells could also form a suppressive shield against immune surveillance.

Endothelial cells can generate tractional forces, appropriate matrices, cord-like tessellated structures because of a limited degree of cell-ECM attachment and a high degree of cell-cell attachment [8]. The invasive cell generates channels consisting of a tubular network embedded in the underlying substrate rather than a tessellation raised above the monolayer. These aggressive cells generate cords similar in appearance to endothelium within the first few hours of culturing, forming patterned tubes which are acellular and which resemble the dimensions and patterning of networks in tumours. Morphologically the substrate may be porous like a well. So the cells fill the well then grow out or fall to spread into another well. Hence the hexagonal type pattern between sprouts. Perhaps the cells are constrained to these shapes or patterns. This is not to say this situation does not occur in nature on a type 4 collagen extracellular matrix bed. Substrate combination with antibodies may reduce this spreading effect.

Endothelial cell shape requirements in the context of growth and limited cell detachment typical of endothelial cell differentiation may be mediated by the mechanical properties of a continuous cytomatix, between the endothelial cell adhesion receptors and its nucleus and could determine the response of the cell to its changing architectural environment via a hard wired signalling mechanism. The extended ECM/cyto-matrix/nuclear matrix as a unit of structure/function is evidenced by comparable tubular structures formed in culture by highly invasive, interconnected primary and metastatic melanoma. These have similarities to cords or tessellations generated by a variety of other cell types including mammary epithelial cells on the appropriate substrate. It is assumed that more invasive cells generate cords similar to endothelium within the first few hours of culturing; patterned tubes completely acellular resemble the dimensions and patterning of networks in tumours [3,8]. Hence the continuous tumour-ECM interaction and traction consequently could contribute to tumour remodelling, metastatic potential and vascuologenetically patterned tumour derived vessels and tumours. The observation of antibody-mediated inhibition of vascular mimicry was an intriguing finding suggesting the possible role of the vascular endothelial factors (either paracrine or autocrine) in tumour vascularisation and entrenchment.

The patterns of microcirculatory vessels have been shown to have a prognostic significance in melanomas with those with the presence of loops and networks doing worse [3,9,10]. However review of vascular patterns formed by tumour cell-generated patterns micro-circulations may exist in tumours from other tissues as well. The angiogenic switch hence is defined by both the tumour cells ability to turn host's blood vessels at a given nexus but also by some changes in aggressive tumour cells that allow them to turn themselves into vessels that could provide micro-circulation. Patterned micro-circulation in culture composed of tubules of an acellular extracellular matrix lined on the outside by tumour cells. A tumour circulation not lined by endothelial cells would play a physiological role in the maintenance and growth of aggressive tumours [8]. Human breast epithelial tumour cells form a disorganised mass undergoing phenotypic reversion to form acini resembling normal breast. Reversion occurs simply by manipulating their surface receptors in three dimensional assays.

In criticism of our current study, the networks formed by the cultured cells do resemble microvascular channels but, fall short of being the compelling evidence of vascular mimicry published by Maniotis et al. [1] in their investigation of ocular malignant melanomas, studying the tumour cells both cultured in vitro and in tumour tissue. Our future study will necessitate further light, electron and immuno-histological microscopy of head and neck tumour tissue to confirm the presence of membrane bounded microvascular channels, lined by tumour cells alone, without endothelial cells. Including examination of the cultured cell networks for PAS matrix or sinusoidal channels or by microinjection of dyes to establish flow patterns.

The **clinical implications **are, however, that there is still too little knowledge of how the many events are coordinated and integrated by the cancer cell, with conspiratorial help by the stromal component of the host. Before drug development can proceed with a legitimate chance of success, significant gaps in basic knowledge need to be filled. A tumour circulation not lined by endothelial cells would play an important physiological role in the maintenance and growth of aggressive tumours. This is observed in breast epithelial tumour cells which form disorganised masses which undergo phenotypic reversion to form acini resembling normal tissue. This reversion occurs simply by manipulating their surface receptors in a 3D assay. This continuous tumour extracellular matrix interaction and traction could contribute to tumour remodelling, its metastatic potential and vasculo-genetically patterned tumour derived abnormal vessels. The tubular structures formed in culture by highly invasive, interconnected primary and metastatic melanoma has similarities to cords or tessellation generated by a variety of other tumour cells on the appropriate substrate. Such vasculogenic triggers and their receptors could be the next target for refined pharmacological interventions in the treatment of metastasis, as important if not more than the recently trialled anti-angiogenic drugs. The important difference may be that they could be more specific and avoid the bystander cross receptor cardiac problems attendant with anti-angiogenic therapy.

## Competing interests

The authors declare that they have no competing interests.

## Authors' contributions

All authors have contributed intellectually and to the writing of this manuscript. All authors read and approved the final manuscript.
